# Correlation of mean apparent diffusion coefficient (ADC) and maximal standard uptake value (SUVmax) evaluated by diffusion-weighted MRI and 18F-FDG-PET/CT in children with Hodgkin lymphoma: a feasibility study

**DOI:** 10.2478/raon-2023-0021

**Published:** 2023-06-21

**Authors:** Nicolas Rosbach, Sebastian Fischer, Vitali Koch, Thomas J. Vogl, Konrad Bochennek, Thomas Lehrnbecher, Scherwin Mahmoudi, Leon Grünewald, Frank Grünwald, Simon Bernatz

**Affiliations:** Department of Diagnostic and Interventional Radiology, University Hospital Frankfurt, Goethe University, Frankfurt am Main, Germany; Division of Paediatric Haematology and Oncology, Hospital for Children and Adolescents, University Hospital Frankfurt, Goethe University, Frankfurt am Main, Germany; Department of Nuclear Medicine, University Hospital Frankfurt, Goethe University, Frankfurt am Main, Germany

**Keywords:** Hodgkin lymphoma, diffusion weighted imaging, apparent diffusion coefficient, MRI, PET/CT

## Abstract

**Background:**

The objective was to analyse if magnetic resonance imaging (MRI) can act as a non-radiation exposure surrogate for (18)F-Fluorodeoxyglucose (FDG) positron emission tomography/computed tomography (PET/CT) in children with histologically confirmed Hodgkin lymphoma (HL) before treatment. This was done by analysing a potential correlation between apparent diffusion coefficient (ADC) in MRI and the maximum standardized uptake value (SUVmax) in FDG-PET/CT.

**Patients and methods:**

Seventeen patients (six female, eleven male, median age: 16 years, range: 12–20 years) with histologically confirmed HL were retrospectively analysed. The patients underwent both MRI and (18)F-FDG PET/CT before the start of treatment. (18)F-FDG PET/CT data and correlating ADC maps in MRI were collected. For each HL-lesion two readers independently evaluated the SUVmax and correlating meanADC.

**Results:**

The seventeen patients had a total of 72 evaluable lesions of HL and there was no significant difference in the number of lesions between male and female patients (median male: 15, range: 12–19 years, median female: 17 range: 12–18 years, p = 0.021). The mean duration between MRI and PET/CT was 5.9 ± 5.3 days. The inter-reader agreement as assessed by the intraclass correlation coefficient (ICC) was excellent (ICC = 0.98, 95% CI: 0.97–0.99). The correlated SUVmax and meanADC of all 17 patients (ROIs n = 72) showed a strong negative correlation of −0.75 (95% CI: −0.84, – −0.63, p = 0.001). Analysis revealed a difference in the correlations of the examination fields. The correlated SUVmax and meanADC showed a strong correlation at neck and thoracal examinations (neck: −0.83, 95% CI: −0.93, – −0.63, p < 0.0001, thoracal: −0.82, 95% CI: −0.91, – −0.64, p < 0.0001) and a fair correlation at abdominal examinations of −0.62 (95% CI: −0.83, – −0.28, p = 0.001).

**Conclusions:**

SUVmax and meanADC showed a strong negative correlation in paediatric HL lesions. The assessment seemed robust according to inter-reader agreements. Our results suggest that ADC maps and meanADC have the potential to replace PET/CT in the analysis of disease activity in paediatric Hodgkin lymphoma patients. This may help reduce the number of PET/CT examinations and decrease radiation exposure to children.

## Introduction

Hodgkin lymphoma (HL) accounts for approximately 6% of all paediatric cancers. It has an incidence rate of 12 cases per million per year in the age group 0–14 with a male predominance.^1,2^ Clinical trials and advances in therapy lead to an improvement of the 5-year survival rate for children newly diagnosed with HL.^3,4^ The current National Comprehensive Cancer Network (NCCN) guidelines do not address HL in paediatric patients.^5^ Therefore, initial radiological staging examinations depend on study protocols. Most patients with HL receive (18)F-Fluorodeoxyglucose (FDG) positron emission tomography/computed tomography (PET/CT) scans as initial staging and during follow-up to assess early response and to identify responders or non-responders to chemotherapy.^6,7,8^ Over 95% of children with HL will become long-time survivors.^4^ Currently the Deauville five-point scale is recommended for FDG-PET/CT-based response assessment in patients with lymphoma. It is a visual scale using mediastinal and liver blood pool FDG-uptake as reference points.^9^ The therapeutic improvements lead to increasing live expectancy and increasing number of dose-intense follow-up examinations with PET/CT. Several studies examined methods to reduce the radiation exposure for paediatric patients in whole body PET/CTs, but FDG-PET/CT is still the preferred examination to evaluate the treatment response of HL patients.^10^ Magnetic resonance imaging (MRI) plays an important role in a wide field of paediatric specialities, ranging from acute trauma to oncology.^11,12,13,14^ In HL patients MRI is used to evaluate soft tissue lesions. In contrast to PET/CT imaging there is no radiation exposure in MRI examinations, which is especially beneficial in paediatric patients. In MRI with diffusion weighted imaging (DWI) apparent diffusion coefficient maps can be calculated. Apparent diffusion coefficient (ADC) maps have been utilized in different setting such as ischemic stroke, heart imaging and differentiation between several types of cancer and cancer detection.^15,16,17,18^ The potential of MRI-derived apparent diffusion coefficient measurements as radiation free surrogate for SUVmax has not yet been evaluated. In the present study, we retrospectively evaluated the correlation between ADC and SUVmax in paediatric patients with HL.

## Patients and methods

This retrospective study was approved by the institutional review board (IRB) of the University Hospital Frankfurt (IRB; 2022-603).

Inclusion criteria were (I) histologically confirmed Hodgkin lymphoma with (II) pretherapeutic MRI and (III) (18)F-FDG PET/CT on the same MRI or PET/CT in (IV) patients < 18 years with a (V) maximum duration between MRI and PET/CT of 30 days.

**FIGURE 1.**
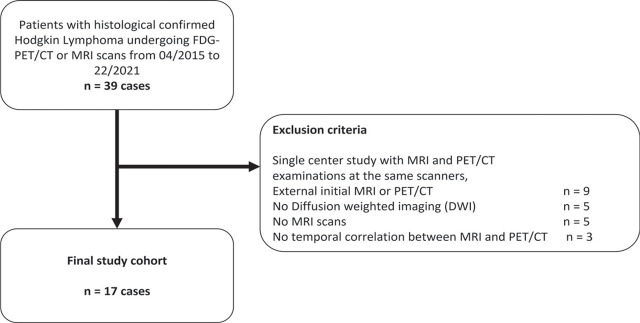
Flowchart for recruitment of study subjects according to the Standards for Reporting of Diagnostic Accuracy (STARD) studies.

Exclusion criteria were (I) missing ADC assessment, (II) duration between MRI and PET/CT > 30 days, (III) imaging artifacts (Figure 1).

### MR imaging acquisition and examination

Examinations of this retrospective single centre study took place at University Hospital Frankfurt am Main/Germany at a single 1.5-T MRI Scanner in clinical routine using a standard 18-channel body-coil (Magnetom Aera; Siemens Healthineers, Forchheim/Germany) and at a single PET/CT Scanner (Biograph 6; Siemens Healthineers, Forchheim/Germany).

Neck MRI examinations were performed using the following sequences: (a) T2-weighted (T2w) Turbo inversion recovery magnitude (TIRM) in transversal orientation, (b) T1-weighted (T1w) turbo spin echo (TSE) in transversal (with fat suppression) and coronal orientation (without fat suppression, substraction images were calculated) with and without contrast media, and diffusion-weighted magnetic resonance imaging (DWI) (b-values: 50, 200, 800).

Body MRI examinations were performed using the following sequences: (a) T2-weighted half-Fourier acquisition single-shot turbo spin-echo (HASTE) in coronal, sagittal and transversal orientation, (b) diffusion-weighted magnetic resonance imaging (b-values: 50, 200, 800), and (c) T1-weighted volumetric interpolated breath-hold examination (VIBE) dixon (with fat suppression) in transversal orientation without breath-hold-imaging, without and with contrast media (Table 1).

**TABLE 1.** Magnetic resonance imaging sequences

**Sequence**	**Orientation**	**Body part**
T2w-TIRM in transversal orientation	transversal	neck
T1w-TSE (fat suppressed, +/− contrast media)	transversal	neck
T1w-TSE (no fat suppression, with substraction, +/− contrast media)	coronal	neck
DWI (b-values: 50, 200, 800)	transversal	neck, body
T2w-HASTE	coronal, sagittal and transversal	body
T1w-VIBE (with fat suppression) without breath-hold-imaging +/− contrast media	transversal	body

DWI = diffusion-weighted magnetic resonance imaging; HASTE = T2-weighted half-Fourier acquisition single-shot turbo spin-echo; T1w = T1-weighted; T2w = T2-weighted; TIRM = turbo inversion recovery magnitude; TSE = turbo spin echo; VIBE = T1-weighted volumetric interpolated breath-hold examination

In PET/CT examinations the mean computed tomography dose index (CTDI) was 2.2 ± 0.8 Milli-Gray (mGy). The mean dose length product (DLP) was 215.1 ± 92.1 mGy*cm (Table 2).

**TABLE 2.** Radiation exposure and examination time

**Modality**	**CTDI [mGy]**		DLP [mGy^*^cm]		**Examination time [min]**	
	Mean (SD)	Range	Mean (SD)	Range	Mean (SD)	Range
FDG-PET/CT	2.2 (0.8)	1.2–4.1	215.1 (92.1)	93.9–410.2	28 (8:26)1	20–49
MRI						
Neck					19:45 (3:41)	17:21–24:47
Thorax					09:23 (2:12)	08:01–10:29
Abdomen					09:23 (2:59)	07:32–12:21

CTDI = computed tomography dose index; DLP = dose length product; mGy = milligray; min = minutes; FDG has to distribute for 1h after application. Time given in the table is the scanning time after application

### Image evaluation

**FIGURE 2.**
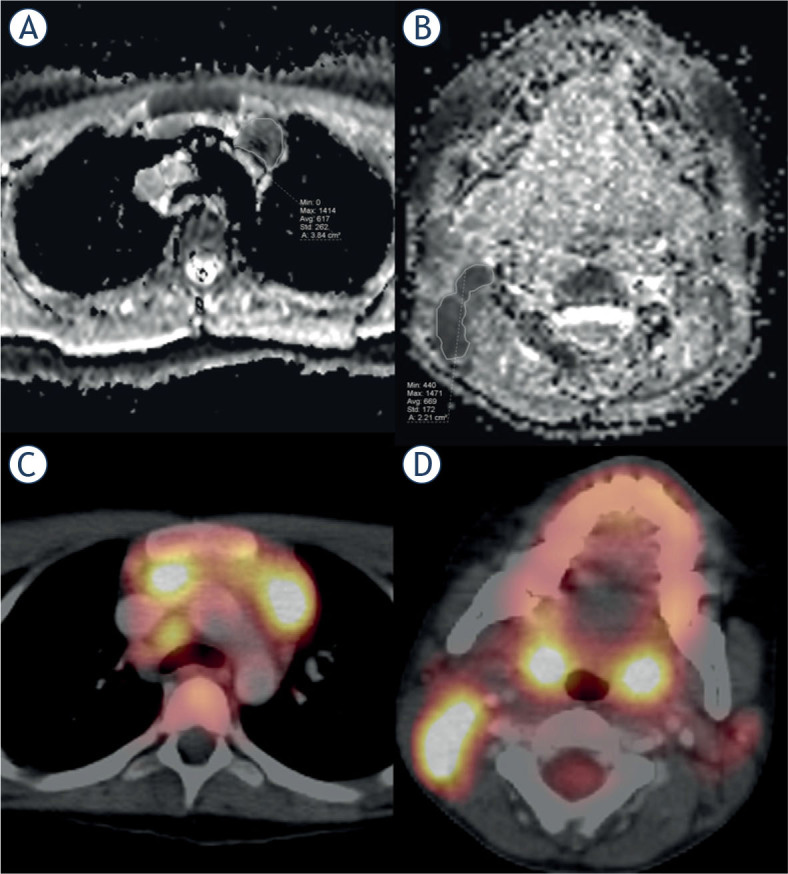
MRI and FDG-PET/CT imaging of two Hodgkin lymphoma (HL) patients. Left side **(A, B)**: 12yo patient with newly diagnosed HL, **(A)** MRI examination with thoracal apparent diffusion coefficient (ADC) map. **(B)** correlating FDG-PET/CT examination with a HL lesion and calculated SUVmax. right side **(C, D)**: 9yo patient with newly diagnosed HL, **(C)** MRI examination with neck ADC map. **(D)** correlating FDG-PET/CT examination with a HL lesion and calculated SUVmax.

Image evaluation was performed by using a conventional picture archiving and communication system station (PACS-station, Centricity Universal Viewer, Version 7.0). MRI examinations and (18) F-FDG PET/CT examinations with temporal correlation (both examinations within one month) were paired (Figure 2). At MRI, the Hodgkin lesions were identified on DWI and ADC. For each Hodgkin lesion in (18)F-FDG PET/CT with measured SUV two readers (N.R, board-certified radiologist with six years of experience and S.B., radiology resident with four years of experience) defined a 2D-ROI in the correlating MRI lesion in ADC maps covering the entire HL lesion and the mean ADC was evaluated. In total 72 ROIs with correlating SUVmax lesions were evaluated. After an initial analysis the 72 ROIs were subdivided into the three different examination areas. 22 ROIs were selected at neck imaging, 28 ROIs at thoracal imaging and 22 ROIs at abdominal imaging. Image quality and noise were evaluated by using a 5-point Likert scales (1, unacceptable; 5, excellent).

### Statistical analysis

Statistical analyses were performed using RStudio 2021.09.2 (Posit PBC). The nonparametric Kolmogorov-Smirnov test was applied to assess the normality of the data. Variables were expressed as means ± standard deviation and analyzed with the Wilcoxon test. A p < .05 (two-tailed) was considered statistically significant. Correlation between SUVmax and meanADC was calculated using the Pearson's Product Moment Correlation Coefficient. The difference between the correlations of neck, thoracal and abdominal meanADC was calculated using the Fisher Z-Transformation with Z Test statistic (Z-Score) and probability (p).^19,20^ According to Landis and Koch, weighted κ statistics was used evaluating the interrater agreement.^21^

## Results

Between April 2015 and November 2021 39 pediatric patients underwent treatment for Hodgkin lymphoma at the University Hospital Frankfurt am Main and received as part of routine diagnostics a PET/CT examination. Out of these, seventeen patients (median age: 16 years, range: 12–20 years; six females [median age: 17 range: 12–18 years] and eleven males [median age: 15, range: 12–19 years]) met the inclusion criteria (Table 3).

**TABLE 3.** Patient characteristics and classifications

**Variable**	**Retrospective cohort of patients diagnosed with Hodgkin lymphoma; baseline features**
No. of patients	17
Median age (SD), years	15.8 (2.2)
Sex	
Male	11 (65%)
Female	6 (35%)
Lugano classification	
1	3 (18%)
2	6 (35%)
3	4 (23%)
4	4 (23%)
Hodgkin lymphoma subtypes (WHO classification)	
Nodular sclerosis	9 (52%)
Mixed cellularity	5 (29%)
Lymphocyte rich	2 (12%)
Lymphocyte depleted	1 (6%)

Unless otherwise indicated, data are the number of patients. WHO = World Health Organization

One ROI was defined in each of the 72 evaluable lesions in MRI examinations of 17 patients (Figure 2).

**FIGURE 3.**
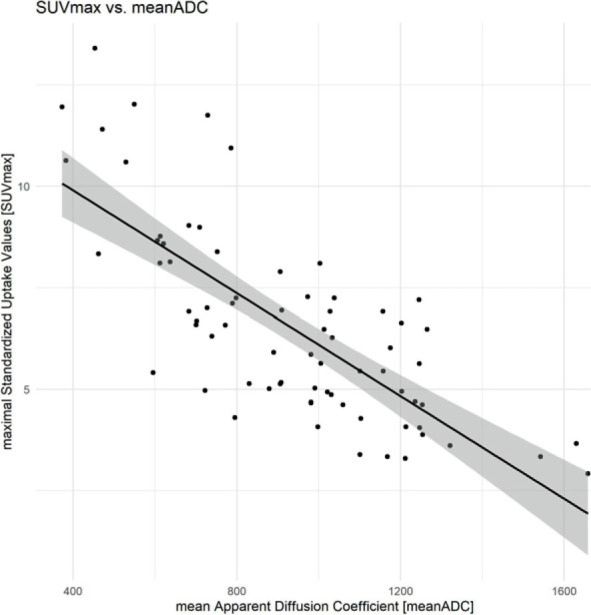
Correlation of SUVmax and mean apparent diffusion coefficient (ADC). The calculated meanADC of the MRI examinations show a strong inverse correlation with the correlating SUVmax of the FDG-PET/CT examinations.

Pretherapeutic mean ADC was 931.17 × 10^−3^ mm^2^/s ± 282.39 × 10^−3^ mm^2^/s (minimum: 373 × 10^−3^ mm^2^/s, maximum: 1658 × 10^−3^ mm^2^/s). Pretherapeutic mean SUVmax was 6.53 ± 2.37 (minimum: 2.92, maximum: 13.4). The meanADC lesions of MRI showed a high inverse correlation of −0.75 (95% CI: −0.84 – −0.63, p = 0.001) with the matched SUVmax (Figure 3) of all 72 ROIs. The intraclass correlation coefficient (ICC) for the evaluation of the mean ADC was 0.98 (95% CI: 0.97–0.99).

The 72 ROIs were then subdivided into 22 neck, 28 thoracal and 22 abdominal lesions.

**FIGURE 4.**
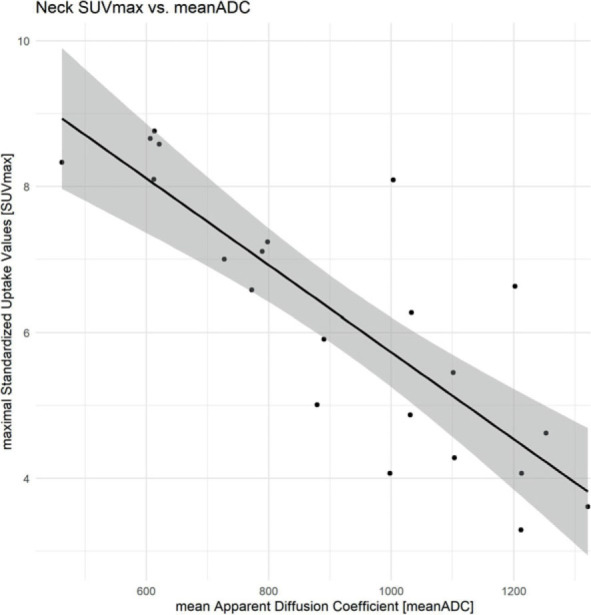
Neck imaging: correlation of SUVmax and mean apparent diffusion coefficient (ADC). The calculated meanADC of the MRI examinations show a strong inverse correlation with the correlating SUVmax of the FDG-PET/CT examinations.

At the neck lesions, pretherapeutic meanADC was 919.95 × 10^−3^ mm^2^/s ± 243.77 × 10^−3^ mm^2^/s (minimum: 462 × 10^−3^ mm^2^/s, maximum: 1321 × 10^−3^ mm^2^/s). Pretherapeutic mean SUVmax was 4.26 ± 0.93 (minimum: 2.85, maximum: 6.04). The meanADC lesions of neck MRI showed a high inverse correlation of −0.83 (95% CI: −0.92 – −0.63, p < 0.001) with the matched SUVmax (Figure 4) of the 22 ROIs. The intraclass correlation coefficient for the evaluation of the neck mean ADC was 0.98 (95% CI: 0.95–0.99).

**FIGURE 5.**
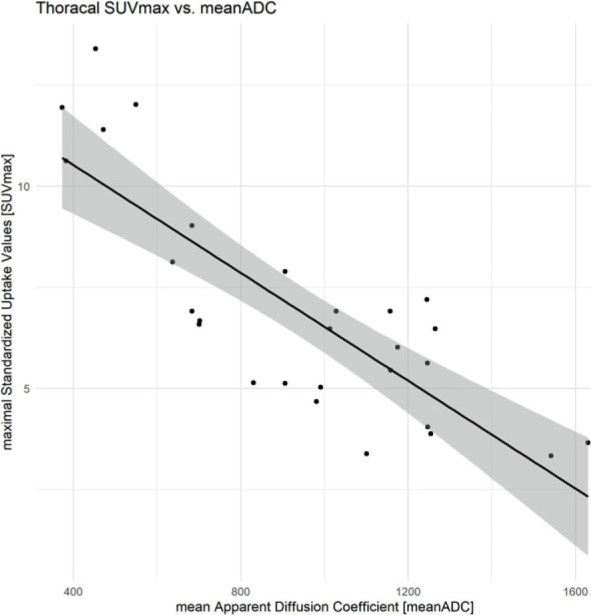
Thoracal imaging: correlation of SUVmax and mean apparent diffusion coefficient (ADC). The calculated meanADC of the MRI examinations show a strong inverse correlation with the correlating SUVmax of the FDG-PET/CT examinations.

At the thoracal lesions, pretherapeutic mean-ADC was 976.22 × 10^−3^ mm^2^/s ± 355.34 × 10^−3^ mm^2^/s (minimum: 373 × 10^−3^ mm^2^/s, maximum: 1630 × 10^−3^ mm^2^/s). Pretherapeutic mean SUVmax was 4.70 ± 1.30 (minimum: 2.82, maximum: 6.94). The meanADC lesions of thoracal MRI showed a high inverse correlation of −0.82 (95% CI: −0.91 – −0.64, p < 0.001) with the matched SUVmax (Figure 5) of the 28 ROIs. The intraclass correlation coefficient for the evaluation of the thoracal mean ADC was 0.99 (95% CI: 0.98–1.00).

**FIGURE 6.**
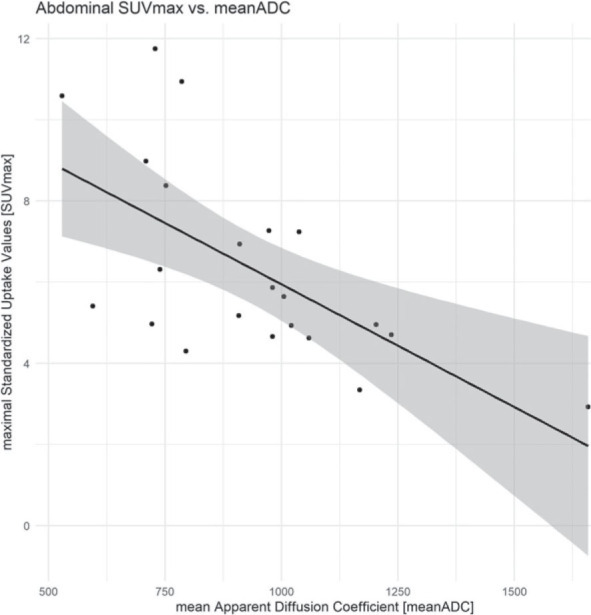
Abdominal imaging: correlation of SUVmax and meanADC. The calculated meanADC of the MRI examinations show a fair inverse correlation with the correlating SUVmax of the FDG-PET/CT examinations.

At the abdominal lesions, pretherapeutic mean-ADC was 931.68 × 10^−3^ mm^2^/s ± 244.72 × 10^−3^ mm^2^/s (minimum: 529 × 10^−3^ mm^2^/s, maximum: 1658 × 10^−3^ mm^2^/s). Pretherapeutic mean SUVmax was 4.66 ± 1.27 (minimum: 2.69, maximum: 7.44). The meanADC lesions of abdominal MRI showed an inverse correlation of −0.62 (95% CI: −0.83 – −0.28, p = 0.001) with the matched SUVmax (Figure 6) of the 22 ROIs. The intraclass correlation coefficient for the evaluation of the abdominal mean ADC was 0.97 (95% CI: 0.95–0.99).

The correlations of neck and thoracal imaging differed not significantly (Z-Score = 0.10, p = 0.92). There is no significant difference of the correlations of neck and abdominal (Z-Score = 1.42, p = 0.15) and of thoracal and abdominal imaging (Z-Score = 1.42, p = 0.16).

### Image ratings

ADC maps were evaluated regarding image noise and image quality. Image noise was rated with mean scores of 4.6 ± 0.7. Image quality was rated with mean scores of 4.4 ± 0.9. The interrater agreement was good for image quality (κ = 0.7 ± 0.14) and image noise (κ = 0.64 ± 0.21) (p < 0.0001).

## Discussion

Currently study protocols for HL patients contain PET/CT and MRI for initial staging, early assessment, and treatment response. Several studies demonstrated the important role of FDG-PET/CT scans as initial staging and during follow-up in HL patients.^22,23,24^ Children are radiation sensitive because of the high cell division rate. Radiation dose induced damages in children are closely examined in several studies.^25^ The increasing number of examinations with X-rays in patients leads to a lifelong increased risk of radiation induced cancer.^26^ Paediatric radiology societies point out the necessity of the ALARA (as low as reasonably achievable) principle in radiation exposure at children.^27^ On the other hand, assessment of the activity by PET/CT might reduce radiation exposure, as patients with negative PET/CT assessed early point during therapy might not receive radiotherapy. MRI might be beneficial in paediatric patients as there is no radiation exposure. Whole-body MRI (WB-MRI) examinations can play an important role as initial staging and follow-up examination in HL patients.^28,29^ Spijkers *et al.* demonstrated a high correlation between WB-MRI with DWI and FDG-PET/CT scans in staging of adult HL patients.^30^ The results of our feasibility study support that the results of Spijkers *et al.* also hold in paediatric patients, as ADC maps and FDG-PET/CT examinations showed a strong inverse correlation.

Our preliminary results in pre-therapeutic imaging suggests that pretherapeutic MRI ADC maps and meanADC demonstrated a strong inverse correlation with SUVmax of FDG-PET/CT neck and thoracal examinations in paediatric HL patients. However, data must be confirmed in the assessment of therapy response.

At abdominal imaging the correlation between meanADC and SUVmax decreased with no significant difference to neck and thoracal imaging. The inter reader agreement at abdominal MRI meanADC was excellent. Noise and image quality did not influence the evaluation of mean ADC. Pediatric MRI examinations were performed without breath-hold imaging. There may be an influence of breathing artifacts on the acquisition of abdominal DWI sequences. Further examinations in breath-hold imaging are necessary to exclude a potential breathing influence.

With this study we shed light on the potential application of MRI instead of PET/CT to assess paediatric patients with HL to reduce radiation exposure.

In this study only pretherapeutic FDG-PET/CT and MRI scans were selected to exclude a potential bias due to treatment. MRI scans with ADC maps may play an important role in follow-up examinations and assessment of treatment response of HL patients. To evaluate a post therapeutic correlation of meanADC and SUVmax further studies are necessary.

The examinations of our study were performed with a single MRI scanner, and one single DWI sequence was used at all patients. This is important as Kivrak *et al.* and Hoang-Dinh *et al*. demonstrated a statistically significant difference in the calculated ADC maps of different MRI scanners from different vendors.^31,32^ The difference in calculated ACD maps may be caused by different DWI sequence settings. Sadinski *et al.* demonstrated a high reproducibility of ADC maps at a single scanner and Newitt *et al.* demonstrated a high reproducibility of ADC maps at different scanners from different vendors with the same DWI settings.^33,34^ The evaluation of the robustness of the pretherapeutic correlation between meanADC and SUVmax in different scanners was beyond the scope of our analysis and requires further studies.

This study has limitations beyond its retrospective design. Missing MRI, missing pretherapeutic MRI and PET/CT examinations and missing temporal correlation between MRI and PET/CT reduced the number of eligible patients, which might has resulted in a selection bias. To exclude inter-scanner noise, we only included examinations from the same scanner. This homogenized the signals, but at the same time, limited the number of eligible patients and might limit the generalizability of the results.
